# Role of LncRNAs in the Epithelial-Mesenchymal Transition in Hepatocellular Carcinoma

**DOI:** 10.3389/fonc.2021.690800

**Published:** 2021-05-25

**Authors:** Xiaoyong Ge, Yuan Yao, Jing Li, Zhaonan Li, Xinwei Han

**Affiliations:** Department of Interventional Radiology, The First Affiliated Hospital of Zhengzhou University, Zhengzhou, China

**Keywords:** long noncoding RNA, hepatocellular carcinoma, epithelial–mesenchymal transition, signaling pathways, biomarker, therapeutic target

## Abstract

Hepatocellular carcinoma (HCC) is a type of primary liver cancer with a high incidence and mortality rate. HCC develops insidiously, and most newly diagnosed cases are in the middle and advanced stages. The epithelial-mesenchymal transition (EMT) is a vital mechanism underlying metastasis in patients with advanced HCC. EMT is a multistep and complex procedure. The promotion and inhibition of EMT directly affect the migration and invasion of HCC. LncRNAs are involved in the epigenetic modification of genes, regulation of gene transcription, and posttranslational modification of proteins. LncRNAs also play important roles in regulating EMT progression in HCC and are promising biomarkers and therapeutic targets. This review focused on summarizing the mechanism by which lncRNAs regulate EMT in HCC. In particular, lncRNAs were reported to primarily act as RNA sponges, and the regulation of EMT involves major signaling pathways. Finally, we reviewed the mechanisms by which lncRNAs are involved in drug resistance and discussed the clinical prospects and potential challenges of utilizing lncRNAs to treat HCC.

## Introduction

Primary liver cancer was the sixth-highest morbidity and third-highest cancer-related mortality worldwide, with 905,677 (4.7% morbidity) new cases and 830,180 (8.3% mortality) deaths according to the Global Cancer Observatory (GCO) (https://gco.iarc.fr/) (1) in 2020. Hepatocellular carcinoma (HCC) is the most common primary liver cancer, accounting for 75-85% of cases (2). The low survival rate of HCC is due to the asymptomatic onset of HCC in the early stage and the loss of the optimal treatment time when it is newly diagnosed in the middle and late stages (3). Middle- and advanced-stage HCC is prone to metastasis, primarily intrahepatic metastasis, which is also a major reason for the poor effect of local treatment or systemic treatment. Epithelial–mesenchymal transition (EMT) plays a vital role in the development, invasion, and metastasis of growing tumors, including HCC (4). Consequently, elucidating the molecular mechanism underlying the role played by EMT in the progression of HCC may contribute to the diagnosis and therapy of cancer and improve the survival of patients.

EMT is a dynamic transformation process driven by induced signals, transcriptional regulators, and downstream effectors (5). Long noncoding RNA (lncRNA), as a regulatory factor, plays an important role in the process of EMT in tumor tissues, which has been revealed in prior research (6). However, there has been no systematic report on the role of lncRNAs in the process of EMT in HCC. This review discusses and summarizes recent work on the mechanism by which lncRNAs affect EMT in HCC.

## Epithelial-Mesenchymal Transition (EMT)

EMT refers to the morphological transformation of epithelial cells into fibroblasts or mesenchymal cells, resulting in the loss of cell polarity, cytoskeletal rearrangement, and increased mobility. Multiple steps, reversibility, plasticity, and heterogeneity are the main characteristics of EMT (7, 8). From the embryonic stage to death, a variety of physiological and pathological EMTs occur in the body (5, 9). EMT is also involved in the pathogenesis of tumorigenesis, progression, metastasis, and drug resistance, which is a kind of pathological EMT (10).

**Figure 1:**
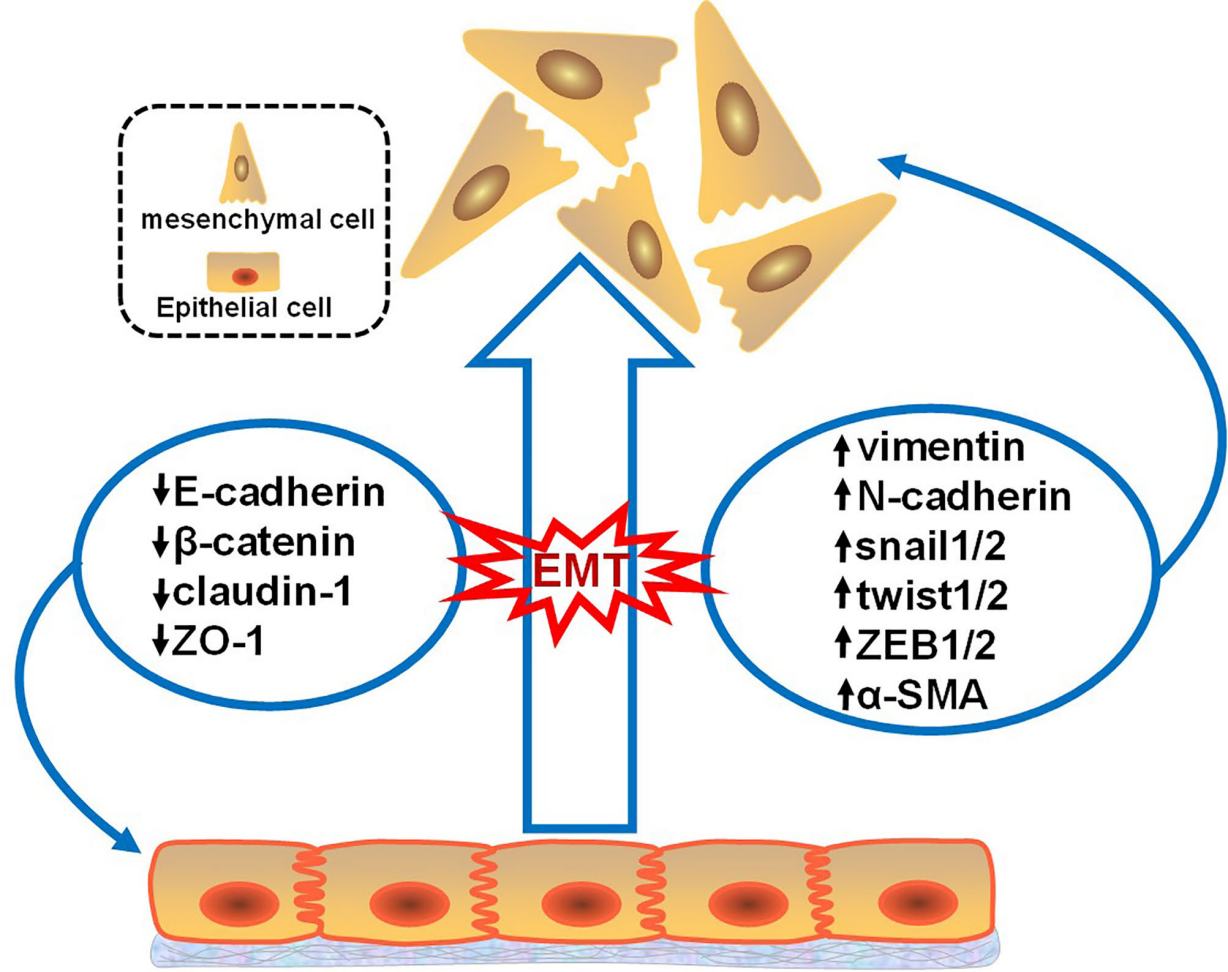
Changes in the EMT markers. This figure illustrates that when tumor epithelial cells develop EMT, epithelial markers decrease and mesenchymal markers increase. Epithelioid cell markers include E-cadherin, β-catenin, claudin-1, and zona occludens 1 (ZO-1). Mesenchymal cell markers include vimentin, N-cadherin, snail1/2, twist1/2, zinc finger E-box-binding homeobox 1 and 2 (ZEB1/2), and α-smooth muscle actin (α-SMA).

The process of EMT involves downregulation of the epithelial cell phenotype and upregulation of the mesenchymal cell phenotype (**Figure 1**). These altered phenotypes are commonly used as molecular markers for EMT; these markers can be divided into epithelioid cell markers and mesenchymal cell markers. Epithelioid cell markers include E-cadherin, β-catenin, claudin-1, and zona occludens 1 (ZO-1), among which E-cadherin is a Ca2+-dependent transmembrane glycoprotein closely related to intercellular adhesion that is mainly distributed on the membrane surface of epithelial cells and is a typical epithelial cell marker. Markers of mesenchymal-like cells include vimentin, N-cadherin, snail1/2, twist1/2, zinc finger E-box-binding homeobox 1 and 2 (ZEB1/2), and α-smooth muscle actin (α-SMA), among which vimentin is mainly expressed in mesenchymal-origin cells, such as endothelial cells, and is considered an important marker of mesenchymal cells (11, 12). Besides, Müller et al. illustrated a key role of iron as a rate-limiting regulator of epigenetic plasticity during epithelial-mesenchymal transition (13).

Transdifferentiation of epithelial cells is performed by EMT-activating transcription factors (EMT-TFs), mainly snail1/2, twist1/2, and ZEB1/2 (11). As mentioned above, EMT-TFs are also markers of mesenchymal-like cells, but they are a special category. EMT-TFs can bind to the E-box proximal to the E-cadherin gene promoter and inhibit the expression of E-cadherin, thereby inducing the EMT phenomenon in cells (14). EMT-TFs can be induced by certain signaling pathways, such as the Wnt/β-catenin signaling pathway, JAK2/STAT3 signaling pathway, MEK/ERK signaling pathway, and PI3K/AKT/mTOR signaling pathway (15). Increasing evidence shows that lncRNAs play a vital role in the regulation of EMT signaling pathways in tumor tissue. For example, lncRNA NR027113 promotes the process of EMT in gastric cancer through the PI3K/AKT signaling pathway (16).

## Long Noncoding RNAs (lncRNAs)

**Figure 2:**
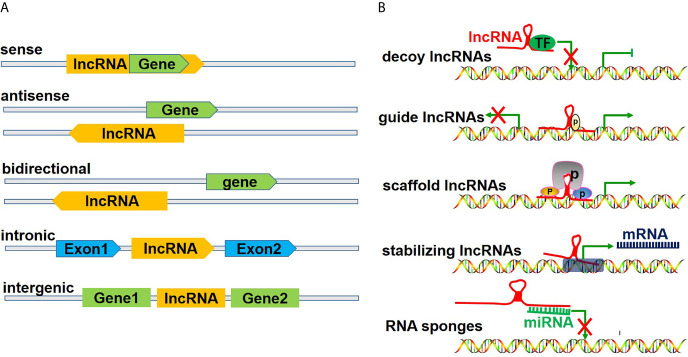
Classification of lncRNAs. **(A)** illustrates the classification of lncRNAs according to the position of lncRNAs in the genome relative to the coding protein gene, including 1) sense lncRNAs, overlapping with coding mRNAs on the coding DNA strand, 2) ant-sense lncRNAs, overlapping with coding mRNAs on the noncoding DNA strand, 3) bidirectional lncRNAs, sharing their transcription initiation sites with noncoding DNA on the contrary strand, 4) intronic lncRNAs, being transcribed from an intronic region of coding DNA, and 5) intergenic lncRNAs, being located between coding DNA. **(B)** shows the classification of lncRNAs according to their functions, including 1) decoy lncRNAs, sequestering proteins to restrict combination with target genes, 2) guide lncRNAs, recruiting chromatin remodeling agents to precise gene loci, 3) scaffold lncRNAs, facilitating protein complex formation, 4) stabilizing lncRNAs, binding target mRNA transcripts and stabilizing and promoting their translation, and 5) competitive endogenous lncRNAs (ceRNAs) or RNA sponges, binding and sequestering miRNAs to restrict their impact on mRNA targets.

LncRNAs, which measure over 200 kb in length, are transcribed by polymerase 2 and have a transcription process similar to that of mRNAs. Previously believed to be merely transcriptional noise, lncRNAs have been increasingly recognized to play critical roles in cellular processes (17). LncRNAs are generally considered functional regulatory factors, but recent studies have shown that lncRNAs can encode certain small peptides and thus play biological roles in a subset of tissues, including tumors (18). According to the position of lncRNAs in the genome relative to protein-coding genes, lncRNAs can be divided into five categories (**Figure 2A**), including 1) sense lncRNAs overlapping with coding mRNAs on the coding DNA strand, 2) antisense lncRNAs overlapping with coding mRNAs on the noncoding DNA strand, 3) bidirectional lncRNAs sharing their transcription initiation sites with noncoding DNA on the contrary strand, 4) intronic lncRNAs transcribed from an intronic region of coding DNA, and 5) intergenic lncRNAs located between coding DNA sites (19). However, as an increasing number of functions of lncRNAs have been discovered, lncRNAs have been classified in another way according to their functions (**Figure 2B**), including 1) decoy lncRNAs, which sequester proteins to prevent them from binding with target genes; 2) guide lncRNAs, which recruit chromatin remodeling agents to precise gene loci; 3) scaffold lncRNAs, which facilitate protein complex formation; 4) stabilizing lncRNAs, which bind target mRNA transcripts to stabilize them and to promote their translation; and 5) competitive endogenous-lncRNAs (ceRNAs), or RNA sponges, which bind and sequester miRNAs to restrict their effects on mRNA targets (20, 21).

LncRNAs are abnormally expressed in tumor cells and play an important regulatory role in tumor proliferation in such cancers as hepatocellular carcinoma (22), breast cancer (23), and gastric cancer (24). In addition, a large number of recent reviews have discussed the role and significance of lncRNAs in the EMT of tumors (6, 20, 25). However, to the best of our knowledge, there has not been a review on the role of lncRNAs in EMT in HCC.

## LncRNAs Involvement in EMT of HCC

Accumulating evidence demonstrates that EMT is a crucial mechanism and link in the development and progression of HCC, especially in the process of migration and invasion (26–28). Because of the stimulation of the tumor microenvironment, the epithelial phenotypes of HCC cells acquire the ability to migrate and invade, facilitating the localization of cancer cells to other tissues and organs or other regions within tissues. This process is often referred to as the epithelial-mesenchymal transition, or EMT. In this process, a series of complex reactions are caused, leading to an increase in EMT-TFs. Upregulated EMT-TFs inhibit the expression of epithelial cell markers and promote the expression of mesenchymal cells. In HCC cells, the silencing or deletion of the common epithelial cell markers E-cadherin and ZO-1 results in the loss of adhesion of epithelial cells and a decrease in cell-cell junction tightness, which can easily leave epithelial cells in a free state. Common markers of mesenchymal cells are N-cadherin and vimentin, among which N-cadherin is transformed from E-cadherin, which can increase the motility of cells and the ability of invasion and metastasis. Additionally, snail1/2, twist1/2, and zeb1/2 are biomarkers of mesenchymal cells and also pivotal transcription factors. In the process of transition of tumor cells, a subset of epithelial cells may not be completely transformed into mesenchymal cells. There are both epithelial and mesenchymal cell markers in the cells, which exhibit an intermediate state, or partial EMT. When transformed epithelial cells migrate to other areas due to their mesenchymal ability, they are stimulated by the local environment, which triggers transformed epithelial cells to re-transform into epithelial cells, thereby facilitating cell proliferation and permanently fixing cancer cells. This process is usually called mesenchymal-epithelial transition (MET) (29–31).

With the development of next-generation sequencing technology (NGS), especially transcriptome sequencing technology (RNA-Seq), a large number of lncRNAs have been identified and determined to be involved in the epigenetic modification of genes, posttranscriptional regulation, protein translation, and modification (21, 32, 33). Since the EMT process involves gene expression and modification, as well as transcription regulation, experts and scholars worldwide are devoting increasing attention to the role of lncRNAs as important regulatory factors in this process. LncRNAs ultimately enhance (34, 35) or inhibit (27, 36) the EMT process by directly or indirectly targeting EMT-TFs or EMT markers. As mentioned above, there are five functional categories of lncRNAs, and the most typical category observed in EMT is RNA sponges or competitive endogenous RNA (ceRNA) (**Table 1**), which can competitively bind with miRNAs and upregulate the expression of target genes to regulate EMT. In addition, lncRNAs may regulate the EMT process through other functional mechanisms, but reports on this topic are relatively scarce, and further research is warranted.

**Table 1:** LncRNAs that act as RNA sponges or ceRNAs to regulate EMT.

LncRNA	Expression status in HCC	FUNCTION	Molecular mechanisms	Effect on EMT	Marker of EMT	Ref
**DANCR**	Overexpressed	RNA sponges	DANCR/miR‐27a‐3p/ LIMK1/CFL1 axis	promoted	E‐cad decreased; N‐cad and Vim increased	2019 (37)
**HOXA11−AS**	Overexpressed	RNA sponges	HOXA11−AS/miR−506−3p/ Slug(snail 2) axis	promoted	E‐cad decreased; N‐cad and Vim increased	2020 (38)
**AC092171.4**	Overexpressed	RNA sponges	AC092171.4/miRNA-1271/ GRB2/ERK/AKT axis	promoted	E‐cad decreased; N‐cad and Vim increased	2020 (39)
**AGAP2-AS1**	Overexpressed	RNA sponges	AGAP2-AS1/miR-16-5p/ ANXA11/AKT axis	promoted	E‐cad decreased; Vim increased	2019 (40)
**SNHG5**	Overexpressed	RNA sponges	SNHG5/miR-26a-5p/GSK-3β axis	promoted	E‐cad and ZO-1 decreased; N‐cad and Vim increased	2018 (41)
**TMPO-AS1**	Overexpressed	RNA sponges	TMPO-AS1/ miR-329-3p/ FOXK1/ AKT/mTOR axis	promoted	E‐cad decreased; N‐cad increased	2020 (42)
**SNHG17**	Overexpressed	RNA sponges	SNHG17/miR-3180-3p/RFX1 axis	promoted	E‐cad decreased; Vim increased	2021 (43)
**H19**	Overexpressed	RNA sponges	H19/miRNA-22/EMT axis	promoted	N‐cad, Vim, and β-catenin increased	2019 (44)
	Overexpressed	RNA sponges	H19/miR-15b/CDC42/PAK1 axis	promoted	E‐cad decreased; N‐cad and Vim increased	2019 (45)
	Overexpressed	RNA sponges	H19/miR-193b/MAPK1 axis	promoted	E‐cad decreased; N‐cad and β-catenin increased	2020 (46)
**DLGAP1-AS1**	Overexpressed	RNA sponges	1)DLGAP1-AS1/miR-26a/b-5p/ IL-6/JAK2/STAT3/DLGAP1-AS1 axis 2)DLGAP1-AS1/miR-26a/b-5p/ CDK8/LRP6/Wnt/β-catenin axis	promoted	E‐cad decreased; N-cad, Vim, and twist decreased	2020 (47)
**LINC00355:8**	Overexpressed	ceRNA	LINC00355:8/miR-6777-3p/ Wnt10b/Wnt/β-catenin axis	promoted	E‐cad decreased; N‐cad and β-catenin increased	2020 (48)
**Linc-smad7**	Overexpressed	ceRNA	Linc-smad7/miR-125b/SIRT6 axis	promoted	E‐cad decreased; N‐cad and Vim increased	2020 (49)
**lncDQ**	Overexpressed	ceRNA	LncDQ/miR-15b-5p/Wnt3A/ β-catenin/EMT axis	promoted	E‐cad decreased; N‐cad and β-catenin increased	2021 (50)
**BACE1-AS**	Overexpressed	ceRNA	BACE1-AS/miR-377-3p/ CELF1 axis	promoted	E‐cad decreased; N‐cad and Vim increased	2021 (51)
**lncRNA** **miR503HG**	Underexpressed	ceRNA	miR503HG/miR-15b/PDCD4 axis	suppressed	E‐cad increased; N-cad, Vim, and Snail-1 decreased	2021 (27)
**PIK3CD−AS1**	Underexpressed	ceRNA	PIK3CD-AS1/miR-566/LAST1 axis	suppressed	E‐cad increased; Vim decreased	2019 (52)
**CASC2**	Underexpressed	ceRNA	CASC2/miR-367/FBXW7 axis	suppressed	E‐cad increased; Vim decreased	2017 (53)
**WWOX-AS1**	Underexpressed	RNA sponges	WWOX-AS1/miR-20b-5p/ WWOX axis	suppressed	E‐cad increased; N‐cad and Vim decreased	2020 (36)
**TUSC7**	Underexpressed	RNA sponges	TUSC7/miR-10a/EphA4 axis	suppressed	E‐cad increased; N‐cad decreased	2016 (54)

Notably, the same lncRNA may play a role in EMT through multiple mechanisms. Overexpression of lncRNA H19 could promote the process of EMT by three pathways: 1) the H19/miRNA-22/EMT axis (44); 2) the H19/miR-15b/CDC42/PAK1 axis (45); and 3) the H19/miR-193b/MAPK1 (mitogen-activated protein kinase 1) axis (46). LncRNA H19 acted as an RNA sponge in these three pathways. In addition, Li et al. demonstrated (55) that overexpression of long stress-induced noncoding transcripts 5 (LSINCT5) could promote the EMT process through two functional mechanisms: 1) LSINCT5, as an RNA sponge, promoted EMT by regulating the LSINCT5/miR-4516/STAT3/Bclxl axis; and 2) LSINCT5, as a stabilizing lncRNA, promoted EMT *via* the LSINCT5/HMGA2 (high-mobility group AT-hook 2) axis.

Taken together, these results indicated that lncRNAs exert their functions through lncRNA-gene interactions, lncRNA-miRNA interactions, lncRNA-protein interactions, and mRNA stabilization (56). More importantly, lncRNAs also require the participation of signaling pathways.

## Signaling Pathways Associated With LncRNAs Regulation of EMT

### LncRNAs Regulation of EMT *via* the Positive Feedback Loop

The positive feedback loop is a special signaling pathway, accurately described as the signal loop, which refers to the cascading reaction of the signal factors and, finally, the activation of the original signal factors to achieve a self-amplifying effect. The formation of a positive feedback loop is conducive to the proliferation, differentiation, migration, and invasion of tumors independent of the external environment, which is a particularly important mechanism for the occurrence and development of tumors. There are positive feedback loops in a wide variety of tumors, such as bladder cancer (57), breast cancer (58), and lung cancer (59). Positive feedback pathways related to lncRNA regulation of EMT in HCC have also been described.

**Figure 3:**
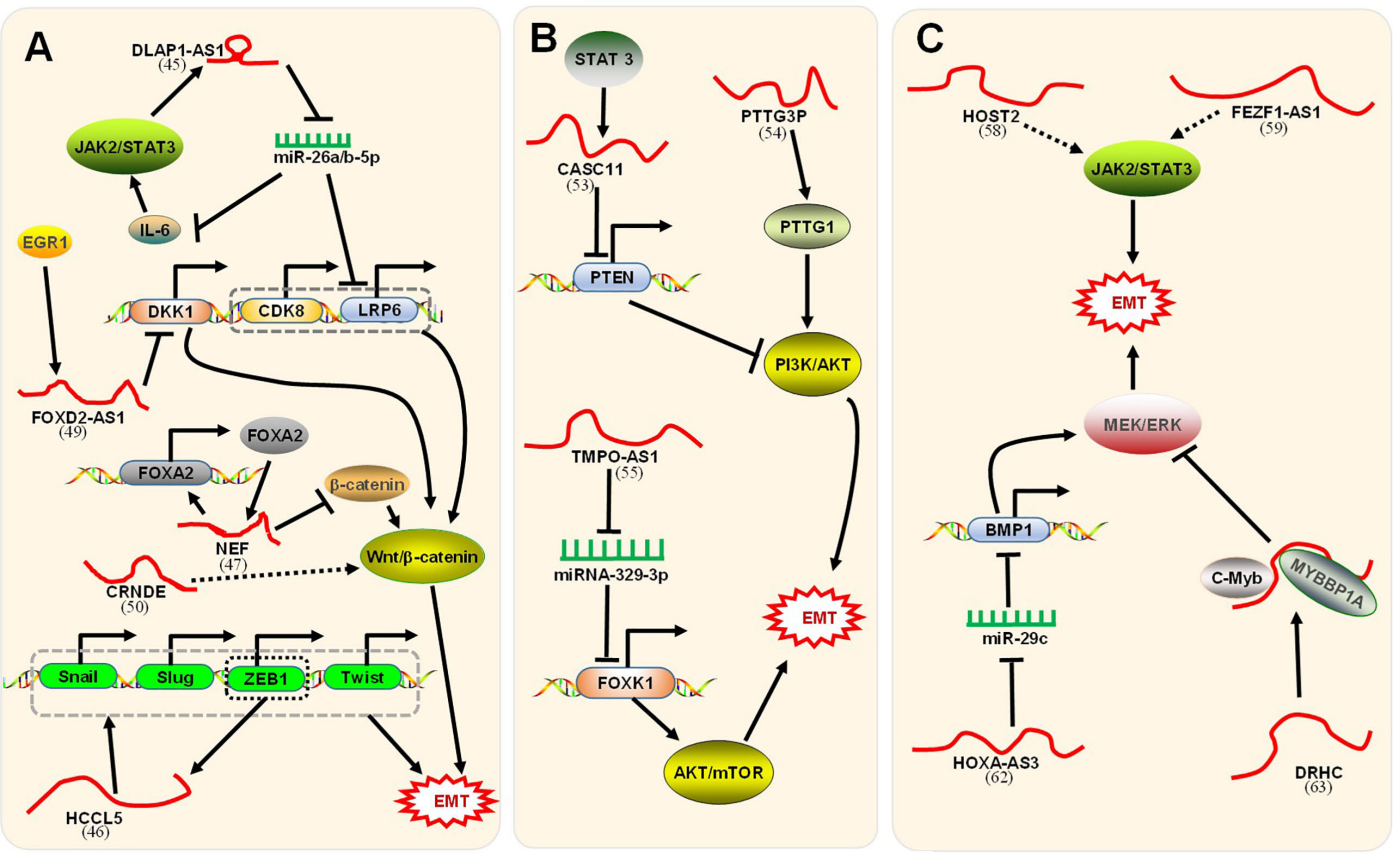
Signaling pathways associated with lncRNAs regulation of EMT. **(A)** The positive feedback loop and Wnt/β-catenin signaling pathway. First, DLGAP1-AS1 (47) promoted EMT through a positive feedback loop. Mechanistically, 1) DLGAP1-AS1 sponged and sequestered miR-26a-5p and miR-26b-5p to upregulate IL-6. IL-6 stimulated the JAK2/STAT3 signaling pathway, which conversely promoted DLGAP1-AS1. 2) DLGAP1-AS1 activated the Wnt/β-catenin pathway by upregulating the downstream CDK8 and LRP6 of miR-26a/b-5p. Second, HCCL5 (60) accelerated EMT by upregulating the expression of Snail, Slug, ZEB1, and Twist1. In turn, HCCL5 was transcriptionally driven by ZEB1. Third, lncRNA-NEF (61) was transcriptionally activated by FOXA2 and, in turn, activated its neighboring gene FOXA2. LncRNA-NEF interacted with β-catenin to increase the binding of GSK3β with β-catenin, leading to the suppression of Wnt/β-catenin signaling. Fourth, lncRNA FOXD2-AS1 (62) induced by EGR1 promoted the EMT process by binding with EZH2 to epigenetically silence the DKK1 gene and activating the Wnt/β-catenin axis. Fifth, CRNDE-promoted Wnt/β-catenin signaling pathway activity was inhibited by CRNDE (63) knockdown. Unfortunately, the paper did not clarify how CRNDE reduces the level of signaling pathway-related proteins. **(B)** The PI3K/AKT/mTOR signaling pathway. First, STAT3-induced lncRNA CASC11 (64) promotes EMT by binding with the enhancer of EZH2 to epigenetically silence PTEN and activate the PI3K/AKT axis. Second, lncRNA PTTG3P (65) promotes EMT by upregulating PTTG1 and activating the PI3K/AKT axis. Third, TMPO-AS1 (42) upregulated the oncogene FOXK1 by sponging miR-329-3p to activate the AKT/mTOR signaling pathway. **(C)** The JAK2/STAT3 signaling pathway and MEK/ERK signaling pathway. First, HOST2 (66) enhanced EMT by upregulating the JAK2-STAT3 signaling pathway. Second, FEZF1-AS1 (67) increased EMT *via* the JAK2/STAT3 signaling pathway. Third, HOXA-AS3 (68) upregulated BMP1 by sponging miR-29c, thus activating the MEK/ERK signaling pathway to enhance EMT. Fourth, the DRHC/MYBBP1A/C-Myb (69) complex with C-Myb inhibited the MEK/ERK signaling pathway and finally inhibited EMT. The corresponding references were also indicated under the lncRNA names on the figure.

LncRNAs can enhance the EMT process through a positive feedback loop (**Figure 3A**). Lin et al. illustrated (47) that the disc large (Drosophila) homolog-associated protein 1 antisense RNA 1 (DLGAP1-AS1) was upregulated in HCC cell lines and promoted the progression of EMT. The regulatory mechanism was divided into two parts. First, DLGAP1-AS1 was an RNA sponge to sequester the HCC-inhibitory miRNAs miR-26a-5p and miR-26b-5p, thereby raising the level of the cytokine interleukin 6 (IL-6). IL-6 stimulated the JAK2/STAT3 signaling pathway and conversely promoted the transcription of DLGAP1-AS1, thereby forming a positive feedback loop. Additionally, DLGAP1-AS1 activated the Wnt/β-catenin pathway by upregulating cyclin-dependent kinase 8 (CDK8) and lipoprotein receptor-related protein 6 (LRP6), downstream elements of miR-26a/b-5p. Lin et al. confirmed (60) that HCCL5, a cytoplasmic lncRNA, accelerated the EMT phenotype by upregulating the expression of transcription factors Snail, Slug, ZEB1, and Twist1 and, in turn, was transcriptionally driven by ZEB1 *via* a superenhancer. In combination, these processes formed a positive feedback loop. Nevertheless, lncRNAs could suppress the EMT process through a positive feedback loop (**Figure 3A**). LncRNA neighboring enhancer of FOXA2 (lncRNA-NEF), a novel lncRNA, was expressed at low levels in HCC. Liang et al. indicated (61) that lncRNA-NEF was transcriptionally activated by EMT suppressor Forkhead box protein A2 (FOXA2) and, in turn, activated its neighboring gene FOXA2. Moreover, lncRNA-NEF interacted with β-catenin to increase the binding of glycogen synthase kinase-3β (GSK3β) with β-catenin and thus promoted the inhibitory phosphorylation of β-catenin, thereby leading to the suppression of Wnt/β-catenin signaling to suppress EMT.

### LncRNAs Regulation of EMT *via* the Wnt/β-Catenin Signaling Pathway

The Wnt/β-catenin signaling pathway refers to the activation of Wnt ligands combined with Frizzled receptors, which triggers a series of signaling reactions and leads to the aggregation of β-catenin in the cytoplasm. Aggregated β-catenin migrates to the nucleus to form nuclear β-catenin, which acts as a transcription cofactor that activates EMT-TFs but is also negatively regulated by E-cadherin (14, 15). The level of β-catenin in HCC cells is regulated by a set of factors, of which lncRNAs are among the important regulatory factors. As mentioned above, in the positive feedback pathway, lncRNA-NEF inhibited EMT by inactivating the Wnt/β-catenin signaling pathway. However, other lncRNAs could stimulate the signaling pathway and promote EMT (**Figure 3A**). As an oncogene, lncRNA FOXD2-AS1 is dysregulated in tumor cells and can be used as a prognostic factor (70). Lei et al. demonstrated (62) that lncRNA FOXD2-AS1 induced by the transcription factor EGR1 promotes the process of EMT by binding with EZH2 (enhancer of zeste homolog 2) to epigenetically silence the gene DKK1 (an inhibitor of the Wnt/β-catenin axis) and activate the Wnt/β-catenin axis. Moreover, Zhu et al. confirmed (63) that colorectal neoplasia differentially expressed (CRNDE), a novel lncRNA, was significantly upregulated in HCC tissue and promoted EMT. *In vitro* cell experiments confirmed that Wnt/β-catenin signaling pathway activity was inhibited by CRNDE knockdown. Unfortunately, this paper did not clarify how CRNDE reduces the levels of signaling pathway-related proteins.

### LncRNAs Regulation of EMT *via* the PI3K/AKT/mTOR Signaling Pathway

The PI3K/AKT/mTOR signaling pathway is relatively common in most cancers. This process involves the phosphorylation and dephosphorylation of related molecules (71). PTEN is considered to be a key negative regulator of the signaling pathway (72). Currently, studies have identified several lncRNAs that function through the PI3K/AKT/mTOR signaling pathway (**Figure 3B**). For example, Han et al. revealed (64) that lncRNA CASC11 induced by STAT3 promotes EMT in HCC by binding with the enhancer of zeste homolog 2 (EZH2) to epigenetically silence PTEN and activate the PI3K/AKT axis. Huang et al. illustrated (65) that lncRNA PTTG3P (pituitary tumor-transforming 3, pseudogene) promotes EMT in HCC by upregulating PTTG1 (pituitary tumor-transforming 1) and activating the PI3K/AKT axis. Moreover, Guo et al. confirmed (42) that abnormal expression of lncRNA TMPO antisense RNA 1 (TMPO-AS1) promotes EMT in HCC. The mechanism in this process was that TMPO-AS1 upregulated the oncogene FOXK1 by sponging miR-329-3p, thereby activating the AKT/mTOR signaling pathway.

### LncRNAs Regulation of EMT *via* the JAK2/STAT3 Signaling Pathway

The Janus kinase 2 (JAK2)/signal transducer and activator of transcription 3 (STAT3) axis has been demonstrated to play a crucial role in the progression of HCC and is involved in the process of tumor metastasis (73, 74). Moreover, lncRNAs regulated the JAK2/STAT3 signaling pathways (**Figure 3C**). Wu et al. proved (66) that human ovarian cancer-specific transcript 2 (HOST2), abnormal regulation of lncRNA in HCC, enhanced EMT by upregulating the JAK2-STAT3 signaling pathway. Cell experiments *in vitro* confirmed that HOST2 increased the expression of EMT-TFs and vimentin by upregulating the protein levels of JAK2 and STAT3 but decreased the expression of E-cadherin. In addition, Wang et al. confirmed (67) that FEZ family zinc finger 1 antisense RNA 1 (FEZF1-AS1), a novel lncRNA measuring 2653 nucleotides in length, was upregulated in HCC and increased EMT. In *in vitro* cell experiments, FEZF1-AS1 knockdown significantly downregulated the JAK2/STAT3 signaling pathway, and JAK2 overexpression significantly reversed the response to EMT inhibition caused by AS1 knockdown. Taken together, these results indicated that FEZF1-AS1, as an oncogene, could promote EMT *via* the JAK2/STAT3 signaling pathway. Furthermore, the JAK2-STAT3 signaling pathway also participated in the above positive feedback loop.

### LncRNAs Regulation of EMT *via* the MEK/ERK Signaling Pathway

Activation of the mitogen-activated protein kinase/extracellular regulated protein kinase (MEK/ERK) axis has been verified to be fundamental in tumor progression (75) and involved in the process of EMT (76). Moreover, lncRNAs regulated the MEK/ERK signaling pathways (**Figure 3C**). Tong et al. demonstrated (68) that HOXA-AS3, a novel lncRNA, was abnormally regulated and stimulated EMT in HCC. HOXA-AS3, as a ceRNA, upregulated the expression of bone morphogenetic protein 1 (BMP1) by sponging miR-29c. BMP1, the downstream target gene of miR-29c, is an oncogene whose overexpression can stimulate the MEK/ERK signaling pathway and promote EMT. These findings indicated that HOXA-AS3 upregulated BMP1 by sponging miR-29c, thereby activating the MEK/ERK signaling pathway to enhance EMT. However, lncRNAs could inhibit EMT by inactivating the MEK/ERK signaling pathway. Zhuang et al. demonstrated (69) that CTC-505O3 (lncRNA DRHC), a novel lncRNA, was underexpressed in HCC and has been shown to inhibit EMT. LncRNA DRHC guided the binding of MYBBP1A and C-Myb to form the lncRNA DRHC/MYBBP1A/C-Myb complex by binding to MYBBP1A. MYBBP1A, a transcriptional coregulator, could bind specifically to and inhibit C-Myb transcription. C-Myb is a proto-oncogene that regulates the MEK/ERK signaling pathway. Taken together, these findings indicated that the lncRNA DRHC bound to MYBBP1A and then formed a lncRNA/DRHC/MYBBP1A/c-Myb complex with c-Myb to inhibit the MEK/ERK signaling pathway and finally inhibit EMT.

### LncRNAs Regulation of EMT *via* Other Signaling Pathways

Ma et al. reported (77) that a novel chromatin-enriched lncRNA, known as metabolism-induced tumor activator 1 (MITA1), induced energy stress, which was upregulated in HCC and conducive to metastasis in the absence of energy. MITA1 accelerated the EMT process largely by increasing Slug (snail2) transcription. In addition, the remaining lncRNAs regulated EMT through various pathways (**Table 2**). LINC00473, IHS, and ANCR were overexpressed in HCC and promoted EMT. However, ELF209 and ID2-AS1 were underexpressed in HCC and suppressed EMT.

**Table 2:** LncRNAs regulate EMT via other special signaling pathways.

LncRNA	Expression statusin HCC	Molecular mechanisms	Effect on EMT	Marker of EMT	Ref
**LINC00473**	Overexpressed	LNC473 upregulates survivin protein by suppressing USP9X-mediated deubiquitination of survivin protein	promoted	E‐cad decreased; N-cad and Vim increased	2018 (78)
**IHS**	Overexpressed	IHS upregulated by HBx-SMYD3 activate PI3K/AKT and ERK signaling by binding and sequestering YBX1 protein in the nucleus, thereby leading to transcriptional activation of MAP3K8 and the instability of DUSP5/DUSP10 mRNA	promoted	E-cad decreased; N-cad and Vim increased	2019 (79)
**ANCR**	Overexpressed	ANCR bound with HNRNPA1 to suppressing its ubiquitination and sponged miR-140-3p to upregulate the expression of HNRNPA1	promoted	E-cad and ZO-1 decreased; N-cad, Vim, and twist1 increased	2020 (80)
**ELF209**	Underexpressed	ELF209 downregulated by HNRNPAB bound and stabilized TPI protein	suppressed	E-cad and ZO-1 increased; N-cad, Vim, and snail decreased	2020 (81)
**ID2-AS1**	Underexpressed	ID2-AS1 upregulated the expression of ID2 by binding with HDAC8	suppressed	E-cad increased; Vim, N-cad, Snail1, and ZEB-1 decreased	2020 (82)

## Conclusion and the Clinical Prospect of LncRNAs

To date, little evidence regarding the clinical application of lncRNAs has been presented. However, studies on the role of lncRNAs in drug resistance and the mechanism of drug effects have been conducted. As an example, lncRNAs were involved in the drug resistance of sorafenib. Zhang et al. proved (83) that the overexpression of small nucleolar RNA host gene 3 (SNHG3) induced EMT and sorafenib resistance by regulating miR‐128/CD151/AKT/PI3K feedback loop signaling. Fan et al. revealed (84) that the overexpression of metastasis associated with lung adenocarcinoma transcript-1 (MALAT1) upregulated Aurora-A expression by sponging miR-140-5p and consequently increasing sorafenib resistance in HCC. Chen et al. illustrated (85) that the overexpression of lncRNA‐POIR accelerated EMT progression and synchronously inhibited sorafenib sensitivity by sponging miR‐182‐5p. Additionally, a large number of agents could inhibit EMT by promoting lncRNA-related pathways. Fan et al. suggested (86) that arsenic trioxide inhibited EMT by promoting lncRNA MEG3 (maternal expression gene 3) upregulation in *in vitro* experiments. Wang et al. showed (87) that melatonin upregulated the transcription of lncRNA-CPS1 intronic transcript 1 (CPS1- IT1) by enhancing the expression of FOXA2, which weakened HIF-1α activity and accordingly inhibited EMT in HCC.

The crucial factors for the poor prognosis of HCC are drug resistance, metastasis, and limited therapeutic targeting options. As mentioned above, lncRNAs were observed to promote EMT and simultaneously enhance sorafenib resistance or reduce sorafenib sensitivity. Consequently, lncRNAs have emerged as promising predictive factors of sorafenib response in HCC. Moreover, lncRNAs can be designed as sorafenib sensitizers to weaken sorafenib resistance and enhance synergistic anticancer effects. EMT is a fundamental biological program of malignant tumor metastasis. In particular, lncRNAs play a key regulatory role in the EMT process of HCC. The expression of lncRNAs that promote EMT is positively correlated with EMT. Hence, lncRNAs may represent an excellent therapeutic target for the treatment of advanced HCC. Additionally, lncRNAs may also serve as new potential biomarkers to predict the prognosis of HCC.

Although the current work is increasingly inclined toward the feasible clinical application of lncRNAs, a number of questions and challenges still exist. First, most of the current studies remain at the basic research stage, primarily at the cellular level, and thus overlook the interaction between cells and the tumor microenvironment. Further clinical trials should be performed to validate the clinical significance of this interaction. Second, with the progress made in sequencing technology, a large number of functional lncRNAs have been identified, and they are gradually increasing. However, the transcriptional amount of each lncRNA may not be uniform in each cancer patient, which may reduce the efficacy of targeted therapy or the sensitivity of lncRNA markers. Third, the upstream regulatory mechanism governing lncRNAs should be further explored. With the progress of science and technology, the utilization of lncRNAs may ultimately enable doctors to make precise clinical decisions from diagnosis to treatment to prognosis evaluation.

In conclusion, the findings described in this review indicate that the regulation of EMT by lncRNAs in HCC is a multistep and complex process that is influenced and regulated by the tumor itself, as well as the tumor environment. With the development of science and technology and the improvement of medical techniques, I believe that the difficulties and challenges associated with this research will be resolved, and lncRNAs will eventually be utilized in the clinic to serve patients.

## Author Contributions

XH, XG, and YY designed the concept of this manuscript. XG, YY, JL, and ZL collected the related paper. XG wrote the manuscript. XH reviewed this manuscript. All authors contributed to the article and approved the submitted version.

## Conflict of Interest

The authors declare that the research was conducted in the absence of any commercial or financial relationships that could be construed as a potential conflict of interest.

## Abbreviations

HCC, hepatocellular carcinoma; EMT, epithelial–mesenchymal transition; PI3K, phosphoinositide-3-kinase; mTOR, mammalian target of rapamycin; AKT, serine-threonine kinase; JAK2, Janus kinase 2; STAT3, signal transducer and activator of transcription 3; ERK, extracellular-signal-regulated kinase; MEK, mitogen-activated protein kinase**;** CFL1, cofilin1**;** E-cad, E‐cadherin**;** N‐cad, N‐cadherin**;** Vim, vimentin**;** ZO-1, zona occludens 1**;** ZEB1/2, zinc finger E-box-binding homeobox 1 and 2**;** α-SMA, α-smooth muscle actin**;** NGS, next-generation sequencing technology**;** RNA-Seq, transcriptome sequencing technology**;** DLGAP1-AS1, discs, large (Drosophila) homolog-associated protein 1 antisense RNA 1**;** CDK8, cyclin-dependent Kinase 8**;** LRP6, lipoprotein receptor-related protein 6**;** lncRNA-NEF, lncRNA neighboring enhancer of FOXA2**;** FOXA2, forkhead box protein A2**;** GSK-3β, glycogen synthase kinase-3β**;** EZH2, enhancer of zeste homolog 2**;** DKK1, dickkopf-1**;** CRNDE, colorectal neoplasia differentially expressed**;** PTEN, phosphatase and tensin homolog**;** PTTG3P, pituitary tumor-transforming 3, pseudogene**;** PTTG1, pituitary tumor-transforming 1**;** TMPO-AS1, TMPO antisense RNA 1**;** FOXK1, forkhead box K1**;** HOST2, human ovarian cancer-specific transcript 2**;** FEZF1-AS1, FEZ family zinc finger 1 antisense RNA 1**;** BMP1, bone morphogenetic protein 1**;** MYBBP1A, MYB Binding Protein 1a**;** MITA1, metabolism-induced tumor activator 1**;** SNHG 3, small nucleolar RNA host gene 3**;** MALAT1, metastasis associated with lung adenocarcinoma transcript-1**;** MEG3, maternal expression gene 3**;** CPS1- IT1, lncRNA-CPS1 intronic transcript 1**;** HIF-1α, hypoxia-inducible factor 1α**;** MAPK1, mitogen-activated protein kinase 1**;** LSINCT5, long stress Induced non-coding transcripts 5**;** HMGA2, high-mobility group AT-hook 2.
